# The study on a new method of preparing PMMA forming composite bipolar plate

**DOI:** 10.1038/s41598-021-88235-2

**Published:** 2021-04-22

**Authors:** Shinn-Dar Wu, Ai-Huei Chiou

**Affiliations:** 1 School of Chemistry and Materials Engineering, Huizhou University, Guangdong, China; 2 Green Energy Research Center, Saint Paul’s College & DePaul University, Lawrenceville, USA; 3 Department of Mechanical and Computer-Aided Engineering, National Formosa University, Yunlin, 632 Taiwan, ROC

**Keywords:** Energy science and technology, Engineering

## Abstract

The recent oil resource shortage has prompted the development of the proton exchange membrane fuel cell (PEMFC) system. PEMFC is a possible source of power that can be used in aircraft, household electricity, agriculture, fishing, motor vehicles, ships, submarines, bicycles, and other portable power systems in the future. This paper emphasizes the production of lightweight bipolar plates to solve several existing problems in the PEMFC system, including weight, cost, and integration. Conventional bipolar plates account for approximately 90% of the weight of battery packs. Therefore, an injection molded flow-field plate constructed from polymethylmethacrylate (PMMA) is developed herein to reduce the weight of the PEMFC system. Computer-aided engineering (CAE) mold flow analysis is then used to simulate the experimental design based on the finished products. Experimental analysis is also performed on the adhesion results of the plates. The results indicate that the establishment of the injection mold using CAE simulation improves mold development and reduces cost. Mechanical coarsening on the surface of the PMMA results in improved adhesion (> 50 N) at temperatures higher than 80 °C. Thus, mechanical coarsening is suitable for the PEMFC system. The problem of conventional weight is solved by reducing the weight by 70%.

## Introduction

**Figure 1:**
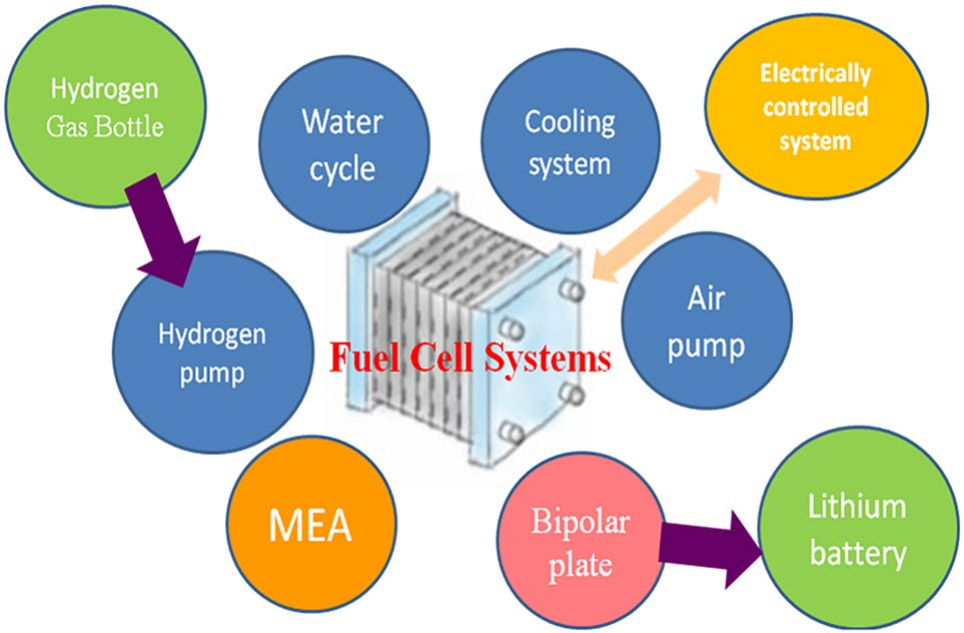
Components of fuel cell system.

The use of fuel cells has changed the way global energy is used. Direct methanol fuel cell (DMFC) and proton exchange membrane fuel cell (PEMFC) will become the core of power generators in the future, and useful power sources for portable equipment, aviation, automobiles, ships, and stationary power systems. However, research on PEMFC has yet to achieve significant breakthroughs. Figure 1 shows that the PEMFC can be classified into the following parts: a proton exchange membrane, catalysts, electrodes, flow field plates, a gas cylinder (or control box), and a lithium battery. Patent protection is also needed to improve and develop the PEMFC. Therefore, further research on this power system is critical. Below are listed the objectives of this paper to improve the design and performance of the flow-field plate of the PEMFC system:

- Bipolar plate materials should be conductive, gas impermeable, heat resistant, and acid resistant. These materials should also have good cooling and water drainage systems and the mechanical strength to support the battery.
- Corrosion-resistant coatings of metal plates require a high budget. The process of producing graphite plates is complicated, and lightweight graphite plates remain unavailable in the market. Therefore, the problems in the production and weight of graphite plates remain unsolved.
- In order to improve the problem of metal and graphite bipolar plates, this research proposes combining surface metal coating and PMMA injection molding production technology.

Buchi et al.^1^ applied the intake fuel without humidity process on graphite, which contained a single battery cell and a small battery group, to produce water indirectly. The researchers succeeded in maintaining the moisture of the surface of the proton exchange membrane, thereby improving the conductivity and self-humidification capabilities of the batteries. Their results showed that the performance of a battery using dry intake fuel increases from 25 to 30% when the temperature of the battery is below 60 °C and the intake fuel is saturated. However, the battery still contains condensed water.

Cho et al.^2^ used the bulk molding compound (BMC) process in composite graphite to produce bipolar plates with a graphite–polymer proportion of 9:1. They found that bipolar plates made with BMC are more electric resistant than those made from injection molding. However, bipolar plates made by injection molding are more durable than those made using BMC. In high-voltage situations, bipolar plates made with injection molding also have better water drainage systems than those made using BMC. Therefore, bipolar plates manufactured with injection molding exhibit better performance than those made using BMC.

Larminie et al.^3^ revealed that a carbon/carbon polymer material can be applied to the bipolar board because the material contains the resin material found on carbon or graphite plate, which can be used to produce bipolar plates by injection molding. Resin graphitization only requires a temperature of approximately 2500 °C. The thickness of bipolar plates should not be lower than 1 mm to avoid plate deformation during the process. Durgasz et al.^4^ noted that the success or failure of the electroless plating process is based on the pretreatment action. Improper pretreatment may result in poor coat adhesion, roughness, and a porous surface. Pretreatment methods vary for different materials. Pretreatment can be broadly classified into two parts: cleaning and activation. Cleaning is an important process that enhances the quality of any substrate.

**Figure 2:**
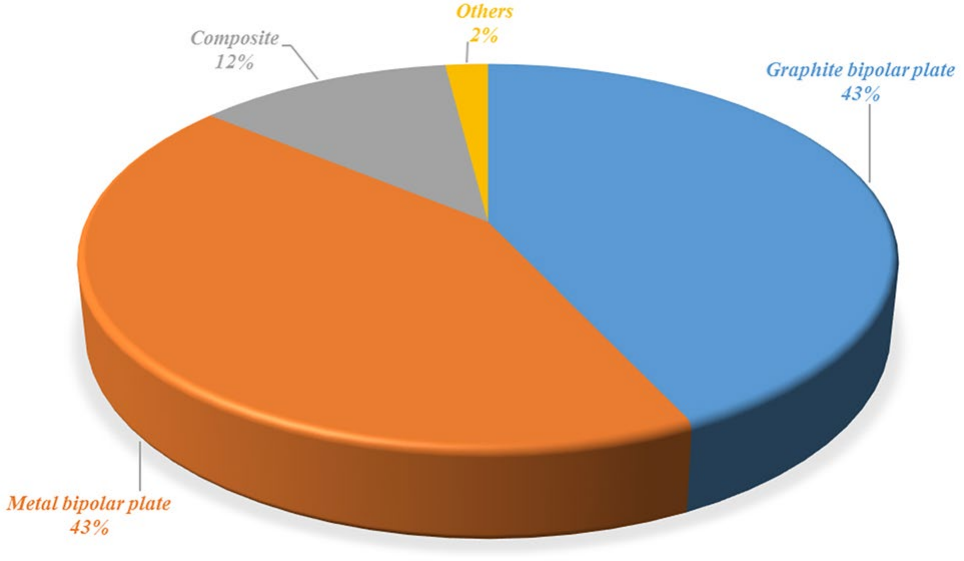
Patent applications for inventions on proportional bipolar plates.

**Figure 3:**
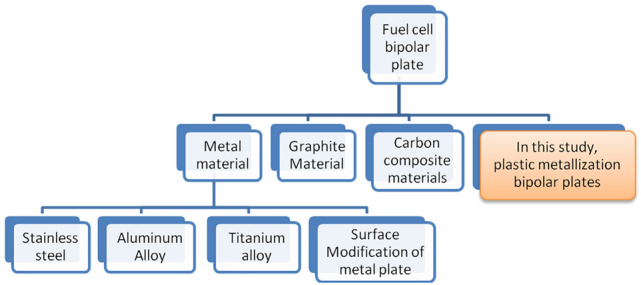
Species of bipolar plates.

**Figure 4:**
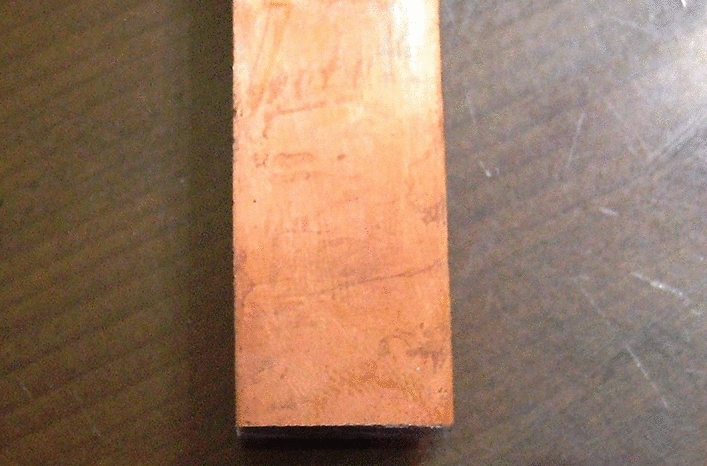
Electrolytic copper and gold PMMA layer.

Based on the proportion of patent applications and the development of fuel cell bipolar plates, it is clear that the development of bipolar plates also affects the overall operation of the fuel cell system of the main components, as shown in Fig. 2. Bipolar plate materials should have isolated gas and mechanical strength and should be conductive as well as heat, acid, water, and corrosion resistant. The classification of bipolar plates is shown in Fig. 3. Pure graphite plate processing is difficult and requires a thick structure because of the processing cost. Dr. Shinn-Dar Wu used composite carbon as bipolar plate in PEMFC, but the surface resistance was too high. Therefore, this study combines the characteristics of these materials to improve mass production, weight reduction, and lower costs. This study focuses on the design and development of bipolar plates of PEMFC and DMFC systems, bipolar plate injection mold design, and CAE mold flow analysis. Metals and plastics are considered the main research focus, as shown in Fig. 4^5^.

Researchers in the materials, preparation, and application research status of proton exchange membrane fuel cell bipolar plates have also in practice modified the stainless steel bipolar plates coated with polyaniline/zinc-porphyrin composite coatings for proton exchange membrane fuel cells. A review of corrosion resistance methods of stainless steel bipolar plates, and evaluation of the effects of metal bipolar plate coatings on the performance of proton exchange membrane fuel cells^6–9^. From the flow channel design and material composite research, comprehensive research on the influence of bipolar plate (BP) geometric design on the performance of proton exchange membrane (PEM) fuel cell is also effective for polypropylene/polypropylene/polypropylene in the presence of carbon nanotubes or graphene as auxiliary fillers. In addition, the fiber orientation effect of carbon fiber composites also has an effect on PEMFC. Researchers have especially explored the influence of the geometric parameters of the flow field on the performance of PEM fuel cells and the high-efficiency composite bipolar plate reinforced with carbon fiber and graphene for proton exchange membrane fuel cells to address the bipolar plate problem^10–13^.

In the study of bipolar plate materials for proton exchange membrane fuel cells, the preparation and performance of 5052 aluminum alloy bipolar plates with electroless nickel plating and gold immersion-polytetrafluoroethylene composite coating are used to address surface corrosion problems, especially with materials with different contact angles. Experimental research on coating bipolar plates to evaluate the performance of PEM fuel cells, the anti-corrosion performance was used of PPY-graphene oxide/PPY-camphorsulfonic acid composite coatings for 304SS bipolar plates in proton exchange membrane fuel cells^14–17^. At the same time, understanding of the influence of compression molding parameters on the electrical and physical properties of polymer composite bipolar plates, the corrosion resistance of nitriding nickel-free stainless steel for polymer electrolyte fuel cell bipolar plates, will contribute to the research field of metal bipolar plates^18,19^.

Exploring graphite clad laminate material issues and metal bipolar plate coating, this research takes PMMA surface coating its focus. Because PMMA is a non-conductive material and has good water resistance, it solves the problem of water resistance of carbon plates. On the other hands, the metal plate has good electrical conductivity, but it has high processing cost and low hydrogen corrosion resistance. Therefore, this study combines the advantages of two characteristics, water resistance, corrosion resistance, and good electrical conductivity. This study aims to reduce the cost, lessen the weight, and address the problems of corrosion resistance and electrical conductivity in bipolar plates. Solving these problems will make bipolar plates more useful in aircraft, submarines, bicycles, and other systems that require portable power in the future.

## Experiment

### Different gate directions design for PMMA bipolar plate by CAE mold flow analysis

**Figure 5:**
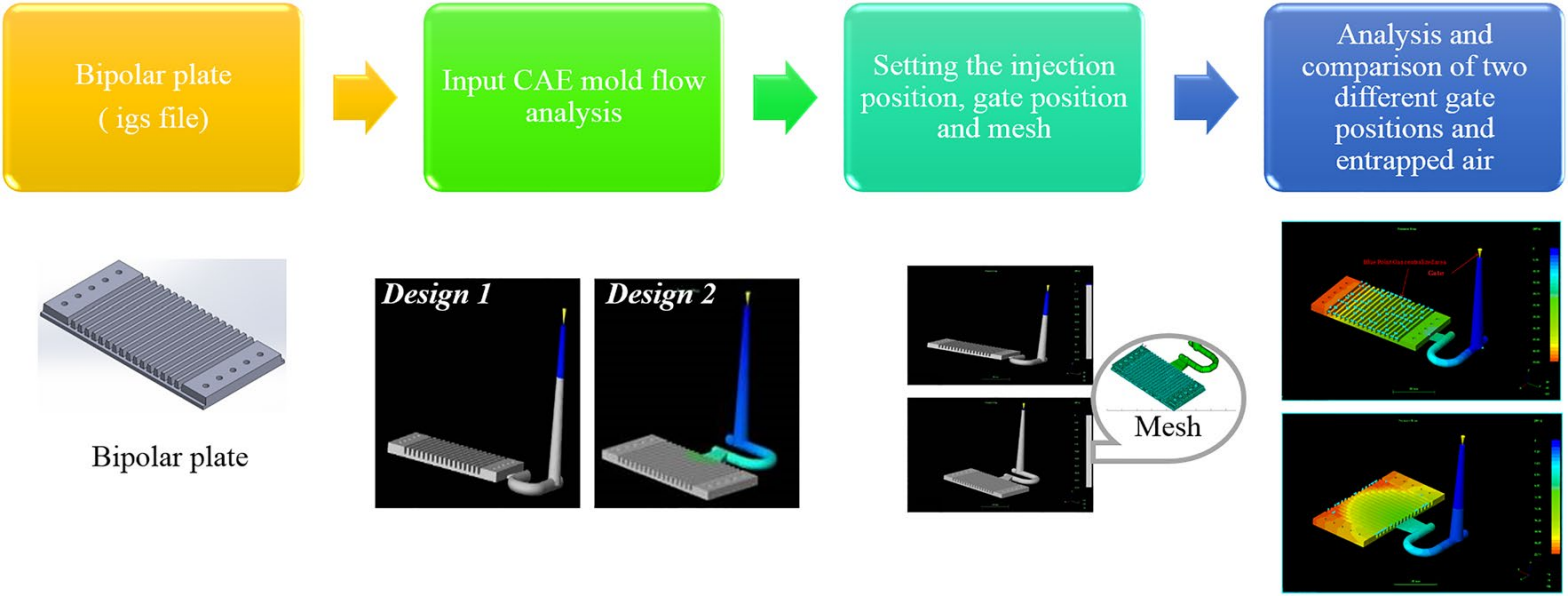
The procedure of CAE mold flow analysis for PMMA bipolar plates with different gate.

**Figure 6:**
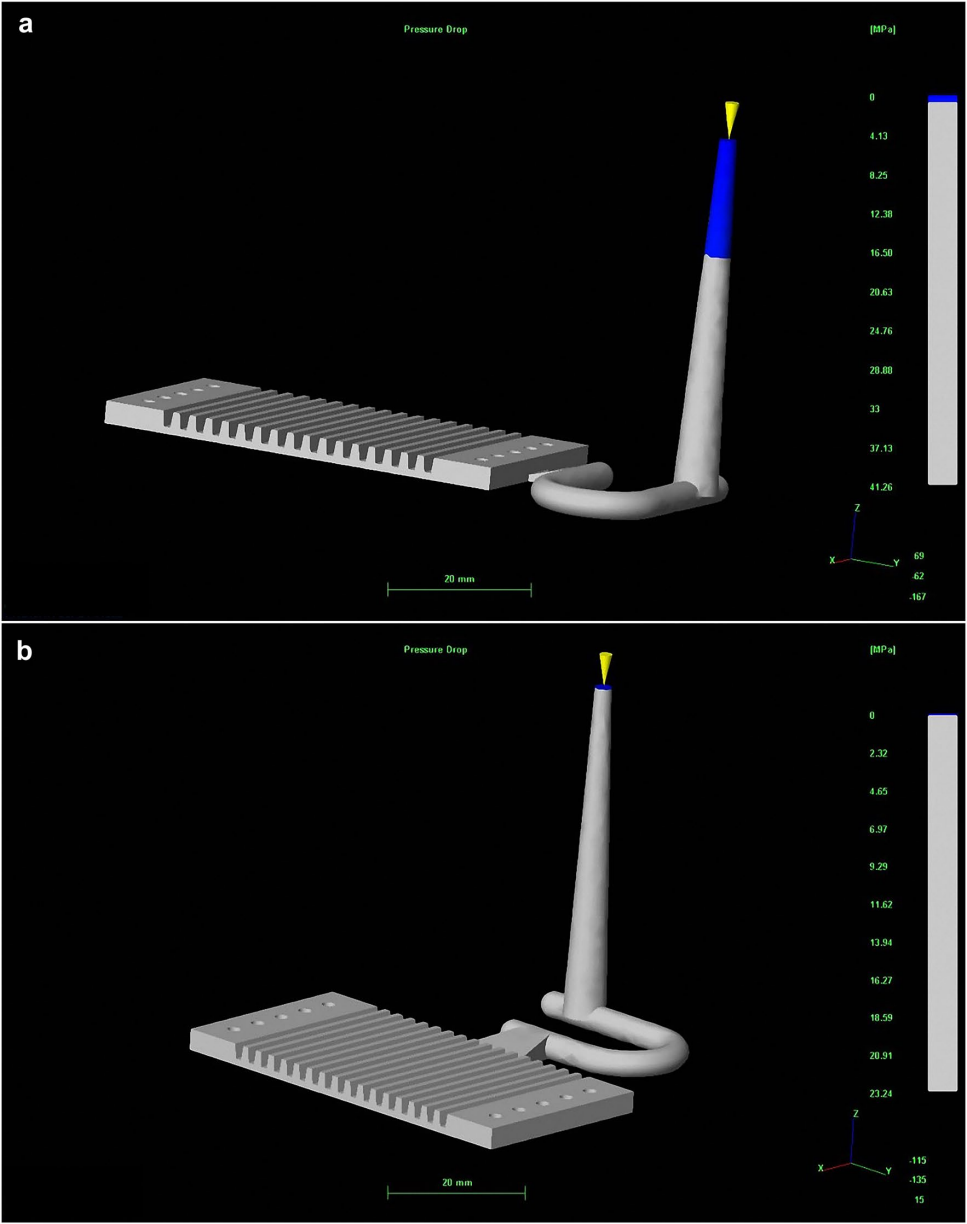
Different entrance directions of PMMA bipolar plate: (**a**) top, and (**b**) side (version number: MOLDFLOW ADVISER 2019, URL link: https://www.autodesk.com/products/moldflow/overview).

The application of CAE analysis in injection-molded plastic part has become common in recent years, especially for part structure design and molding process optimization. Bipolar plates plays an important role in fuel cells, especially for the creation of side-flow channels on bipolar plate substrates. Therefore, a design with two different gate directions for the PMMA bipolar plates is discussed in this paper. The CAE analysis uses PMMA bipolar plate for the mold development. The procedure of the CAE mold flow analysis for PMMA bipolar plates with different gates is shown in Fig. 5. The whole process from design and development to sample production uses 3D computer aided drawing. CAE is used to analyze and rectify mold flows in the process of manufacturing molds. A rectangular plate of 20 mm × 50 mm mm molded with PMMA is simulated to validate the prediction of orientation. Figure 6 shows the PMMA bipolar plate geometry and the different gate directions.

### PMMA bipolar plate of mold development

**Figure 7:**
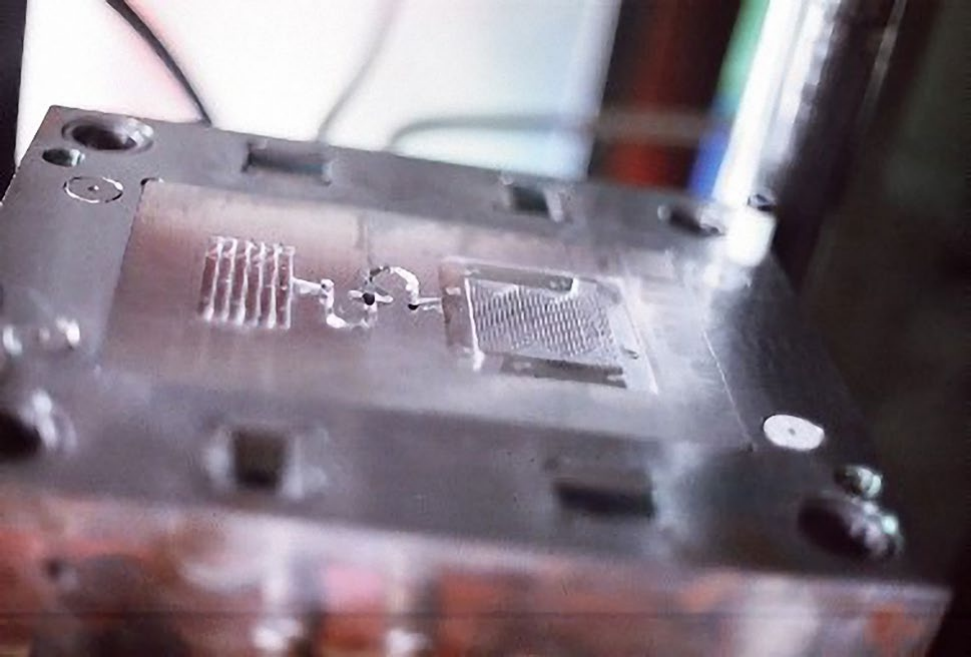
PMMA bipolar plate of Injection mold figure.

Injection molding processes allow cost-effective, large-scale manufacturing of bipolar plates. Therefore, using an injection molding process, PMMA bipolar plates with a total thickness of 3.2 mm and an active area of 20 mm × 50 mm were fabricated. Based on analysis of the mold flow, the actual PMMA bipolar plate shape of the mold was fabricated as shown in Fig. 7. The mold shape and structure of PMMA bipolar plate, that geometry design of a toothed ladder structure with mutually perpendicular directions. Moreover, the mold structure of PMMA bipolar plate include the top design of the bipolar plate board retreat mold.

**Figure 8:**
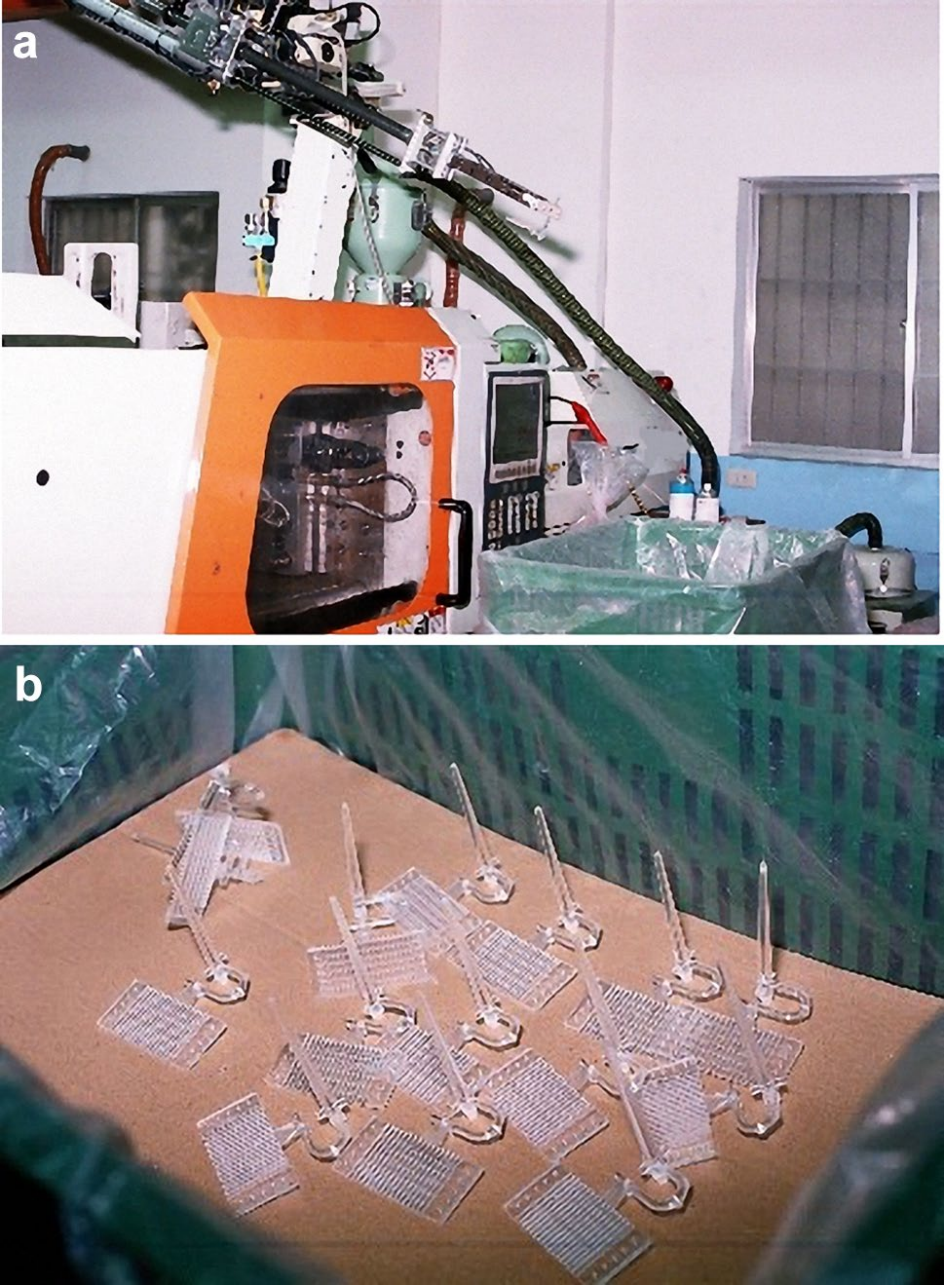
Injection molding machines and PMMA bipolar plate by injection molding.

The PMMA bipolar plate was made by injection molding machine with 50 ton clamping force and mechanical arm, as shown in Fig. 8a. Bipolar plates were pre-dissolved in plastic and then subjected to the injection molding process. The PMMA bipolar plates were prepared with injection pressure of 1185 kgf/cm^2^(about 1162 bar), injection speed of 28 cm^3^/s, holding pressure of 5 s and tube temperature of 205 °C.The bipolar plates made by injection molding were packed and then left to cool for approximately 30 s. They were then removed using the automated mechanical arm. The finished product is shown in Fig. 8b.

### Surface modification of PMMA bipolar plate

Bipolar plates are a key component of PEMFC as they account for significant fractions of their weight and cost. To increase the corrosion resistance and interfacial contact resistance of the PMMA bipolar plates, surface modification was systematically used in their preparation. In the actual measurement of traditional composite carbon bipolar plate, the resistance is between 25 and 35 Ω, and the surface anti-corrosion electroplating of the metal plate is greater than > 1 μm after processing the flow channel. This study combines two characteristics, taking the conductive advantage of the metal plate and removing the carbon weight of the composite carbon plate, so PMMA is used to improve the surface and metal coating, therefore, it has good performance in weight, volume and adhesion of metal coating. Thus, metal-based coatings prepared by surface modification are analyzed in this paper. Before surface modification of the PMMA bipolar plates, the molecular dynamics of the mechanical surfaces were explored. To further understand the deposition mechanism of metal-based coatings, the main purpose of this paper is to study the growth behavior of PMMA in the process of metal deposition, and to simulate the changes in microstructure using three-dimensional molecular dynamics.

This research strengthens the destructive power of the unstable layer, forming a BPSD (Bipolar Plate Surface liquefaction Design, Shinn-Dar) structure on the PMMA material. This enables the metal solid to be better cured on it, which can increase the bonding strength of the metal on the PMMA. After the BPSD structure of the metal surface was constructed, the copper–gold composite film is plated through the conductive layer. During the mechanical roughening of the flow field plates, the surface of the gold film thickened by about 1 μm, thereby improving the performance of the current conduction of the films.

### PMMA bipolar plate characterizations

Weight were recorded after a single PEMFC assembly in using the PMMA bipolar plates by injection mold process. Three watt hour meter, tensile testing machine and potentiostatic instrument were used to investigate electrical conductivity values ,surface adhesion and corrosion situation . Besides, the operating temperature of the PEMFC can be as high as 120 °C, the adhesion force at different temperatures were be discussed. A single cell of PEMFC was assembled with commercial membrane electrode assembly (MEA), SGL gas diffusion layer made out of woven carbon fiber cloth, and created PMMA bipolar plates by injection mold. The MEA with 183 µm of membrane thickness used in the single-cell was produced from Nafion 117 coated by 4.0 mg/cm^2^ of platinum–carbon at an anode side, 4.0 mg/cm^2^ of platinum–carbon at a cathode side. The performance of the single-cell was evaluated by measuring the I–V characteristics using an electronic load.

## Results and discussions

### CAE moldflow and actual product discussions of PMMA bipolar plate

An air trap is air that is caught inside the mold cavity. It becomes trapped by converging polymer melt fronts or because it failed to escape from the mold vents, or mold inserts, which also act as vents. Entrapped air will result in voids and bubbles inside the molded part, incomplete fill, or surface defects such as burn marks or blemishes. Therefore, avoiding air traps inside the mold cavity is an important issue. Re-design the gate and delivery system can decrease entrapped air.

**Figure 9:**
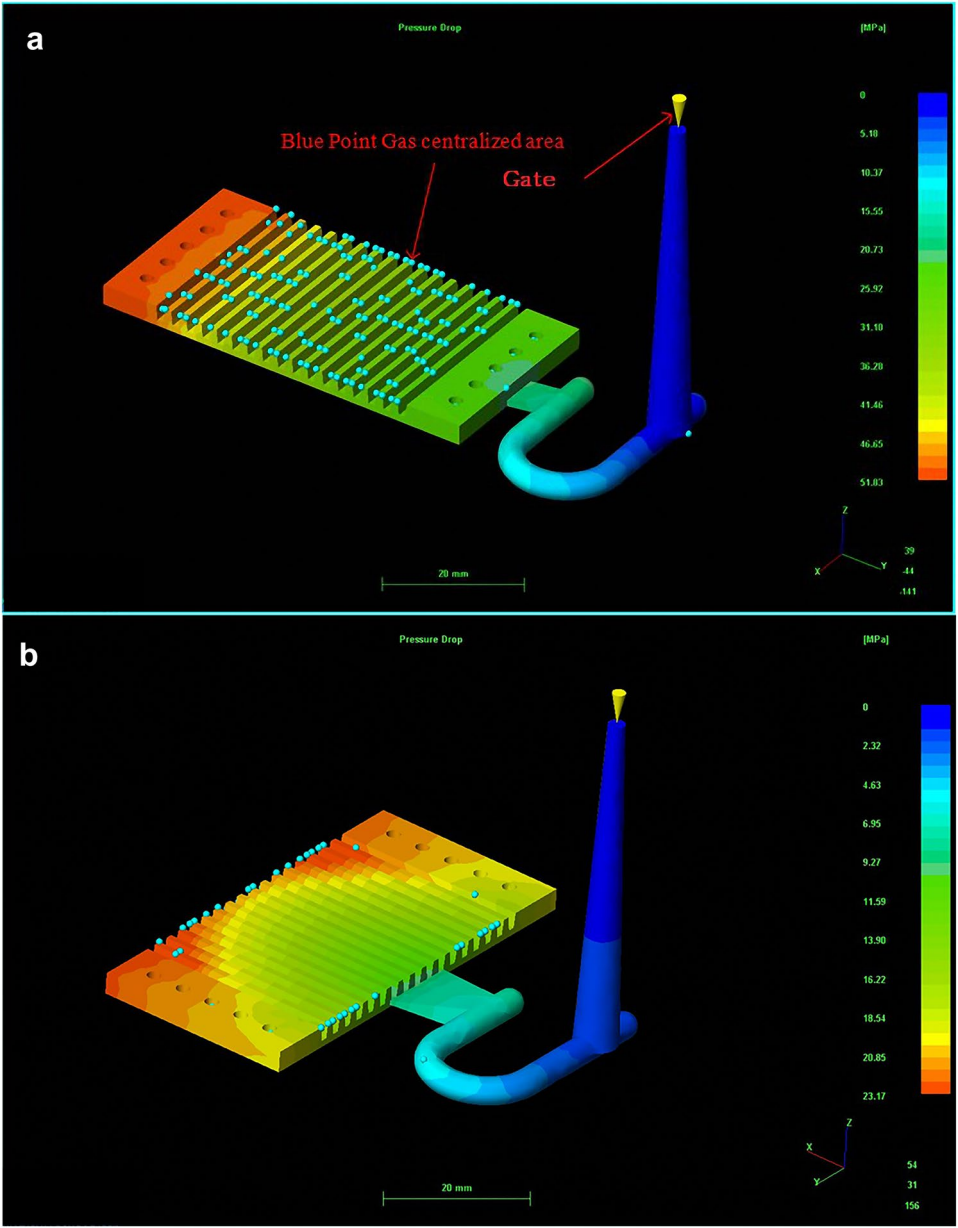
Air traps in injected PMMA bipolar plate for two different design: (**a**) top and (**b**) side (Version number: MOLDFLOW ADVISER 2019, URL link: https://www.autodesk.com/products/moldflow/overview).

The simulated air traps in injected PMMA bipolar plate for two different design were shown in Fig. 9. The numbers of air traps for top design were 154, which were more than that of the side entreated gate design (about 35, Fig. 9b). The more air trap can lead to defects. In this study, the optimal inlet position and the stomata position was the side entrance as shown Fig. 9b. According to the results of CAE mold flow, the actual mold gate adopts side entry for PMMA bipolar plate of the mold were fabricated (Fig. 7). The PMMA bipolar plates were fabricated by injection mold process as shown in the Fig. 8b.

### Surface modification of PMMA bipolar plate

**Figure 10:**
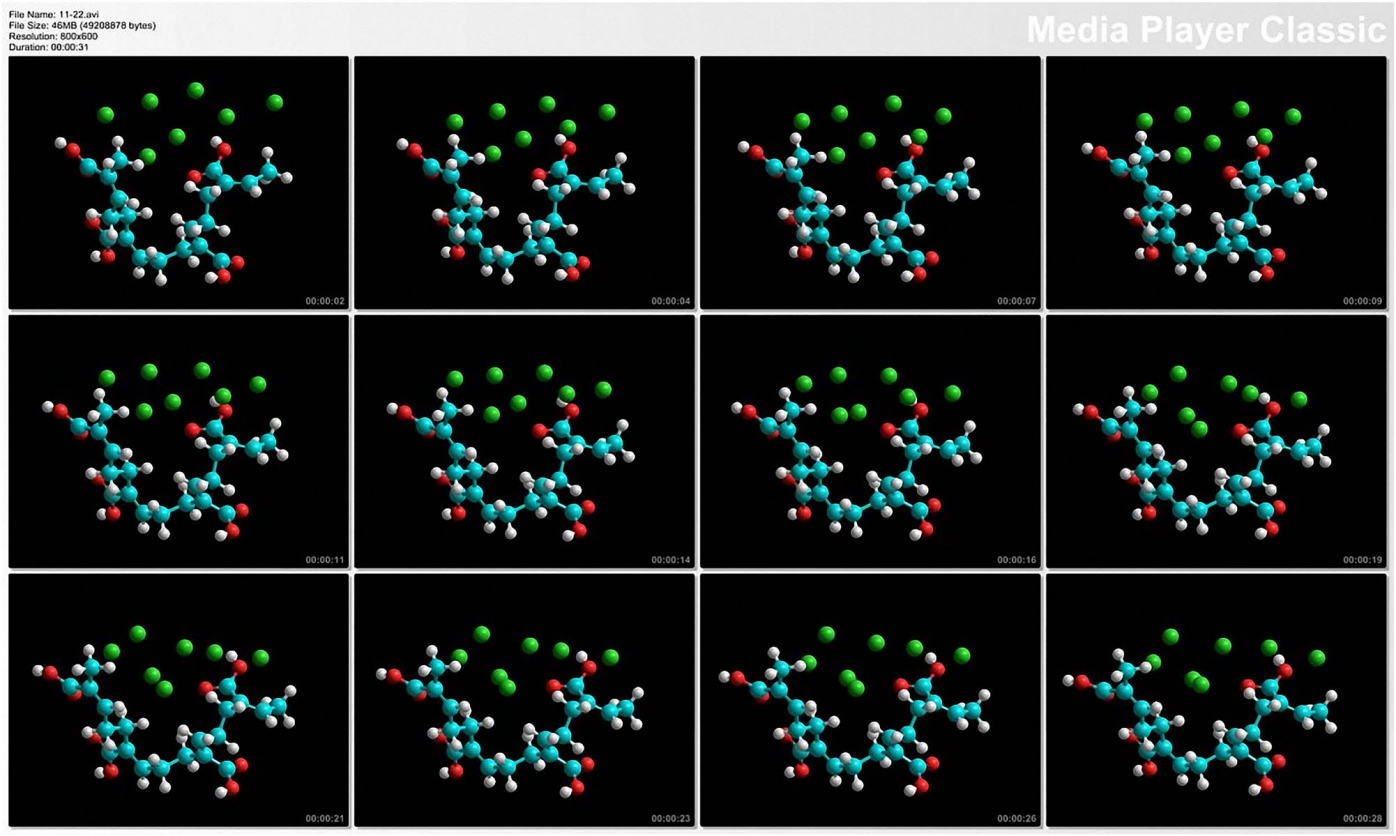
Surface of the molecular dynamics mechanical schematic (O: oxygen in red; C: carbon in blue; H: hydrogen in white; Cu: copper in green) (Version number: HyperChem.v8.0.5).

Molecular dynamics is an indispensable computational tool for studying the microstructure and surface morphological evolution of coatings and thin films upon varying incident flux compositions. The molecular dynamics model shows the PMMA molecular structure, where the long chain-shaped molecular structure formed by the mechanical force of the surface morphology is not flat. The copper deposited on the surface of the binding mode illustrates the different types of copper deposited under the surface conditions, as shown in Fig. 10. The surface roughened by mechanical pretreatment modified the irregular patchy distribution of films. The coatings were deposited through stacking.

In vapor phase lamination or semiconductor lamination, for polymer bonding, oxygen ions (O^–^) are used to destroy the surface of the polymer, so that the polymer surface forms an unstable bond-breaking layer, and the metal surface and the unstable layer form an attractive force. This research strengthens the destructive power of the unstable layer, forming a BPSD (Bipolar Plate Surface liquefaction Design, Shinn-Dar) structure on the PMMA material, which enables the metal solid to be better cured on it, which can increase the bonding strength of the metal on the PMMA.

After the BPSD structure of the metal surface is constructed, the copper–gold composite film is plated through the conductive layer. During the mechanical roughening of the flow field plates, the surface of the gold film thickens by about 1 μm, improving the performance of the current conduction of the films. The average resistance values decreased to 8 Ω using a similar metal conductor. The mechanically roughened surface has an effect on the thickness of the middle layer through the rise and fall of the rate based on a product size of a 20 mm × 50 mm entity to measure the flow field plate resistor value, as shown in Table 1.

**Table 1:**
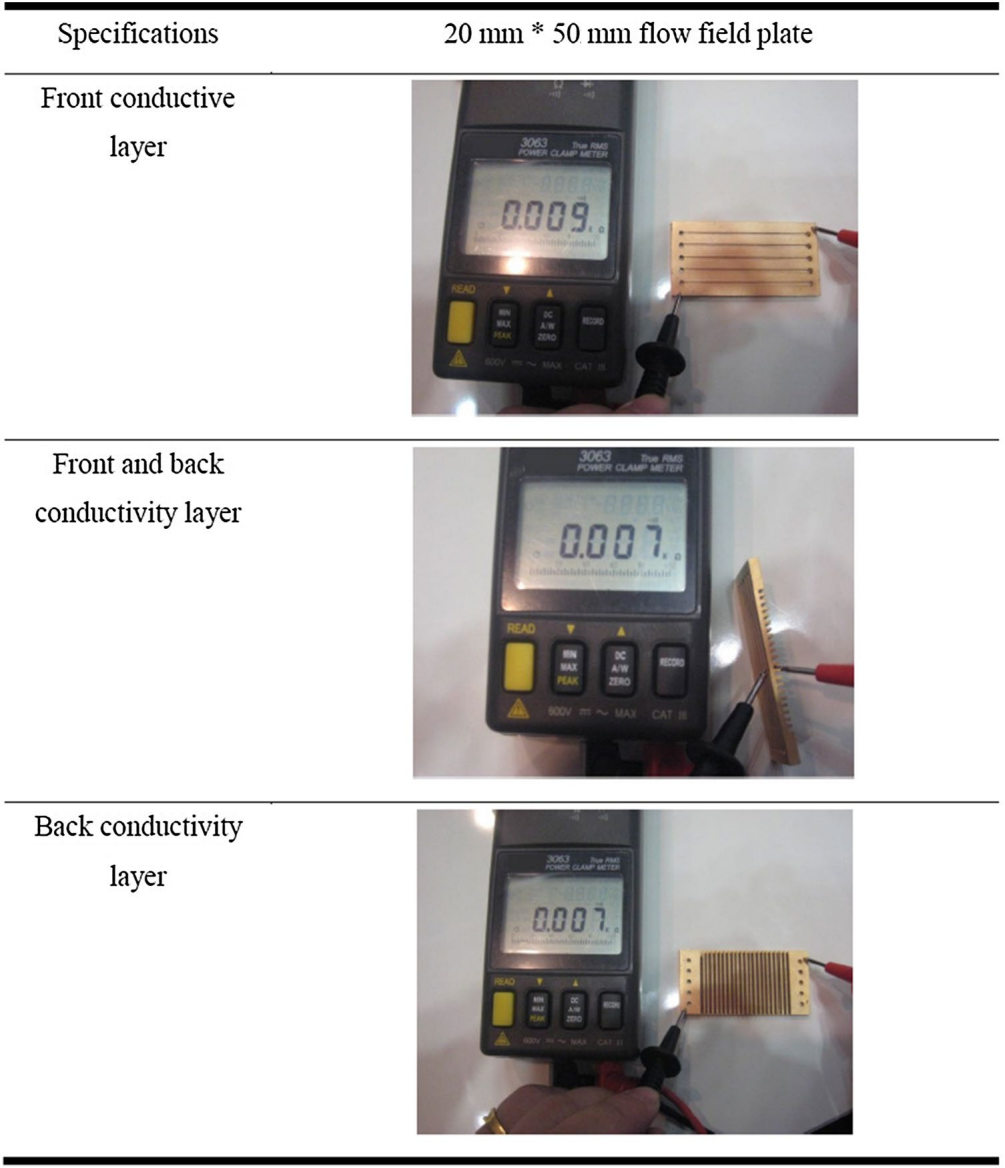
Finished size of 20 mm × 50 mm entity to measure the flow field plate resistor value.

### The adhesion force analysis at different temperatures of PMMA bipolar plate

**Figure 11:**
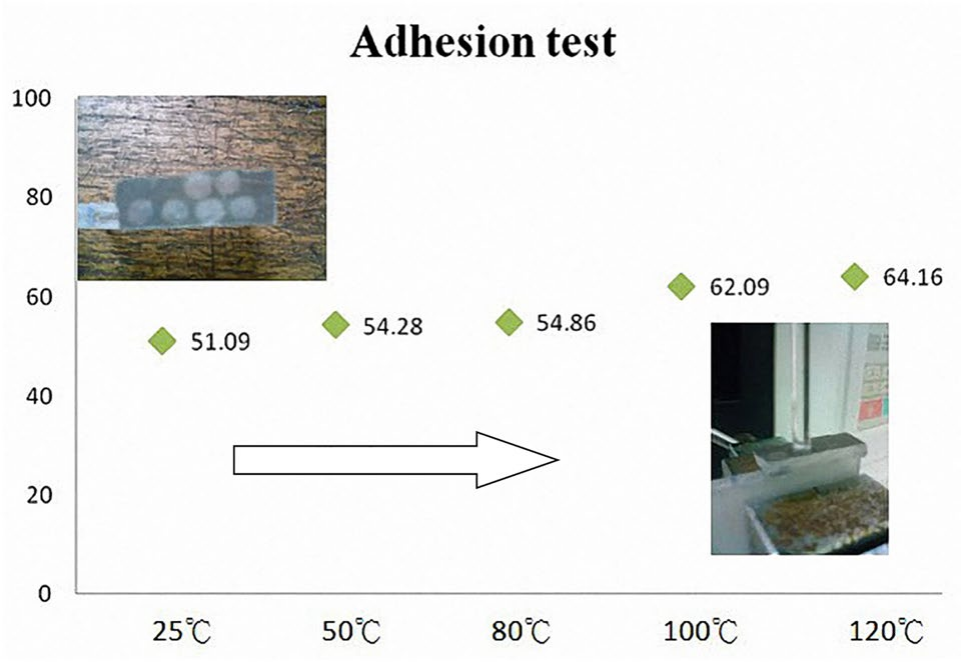
Adhesive comparison (unit: N) at different temperatures. Conventional coating adhesion at 40 N or less.

The temperature of PEMFC can reach 120 °C during operation, and the film were be detached due to thermal expansion. In addition, the coating was easy to be damaged by the post processing of the runner. Therefore, the adhesion force at different temperatures were be discussed in this section. The surface of the bipolar plate was analyzed using the copper vacuum evaporation technique and a film thickness of 3000 Ả. Electroless plating gold and a film thickness of 1 μm were used for post-processing. Tensile tests were performed at a speed of 1 mm/min between the PMMA and the metal layer. The experiment was repeated three times for each set of test pieces, and the average was obtained using the arithmetic mean of the tensile value. Figure 11 shows the different temperatures for the adhesion values, ranging from 50 to 65 N. The maximum forward adhesion value of the plated film minimally changed when subjected to varying temperatures of 25 °C, 50 °C, and 80 °C. However, the adhesion value improved when the plated film was subjected to temperatures ranging from 100 and 120 °C. When the material is at the reaction temperature, there will be thermal expansion and contraction effects, the surface of the PMMA substrate will soften, and then restore its original hardness when cooled. After cooling, the adhesion between the coating layer and the PMMA substrate becomes stronger. This time, the innovative point of this research is to improve the adhesion between the film and the substrate. Especially in the post-processing of traditional coating, the milling cutting force does not exceed 40 N, and the coating is easily damaged. In this study, it was increased to 50 N to 65 N. This study shows that the positive adhesion of the PMMA metal plated film can resist the milling cutting force and resistance to film shedding due to thermal expansion.

### Corrosion analysis of PMMA bipolar plate

**Figure 12:**
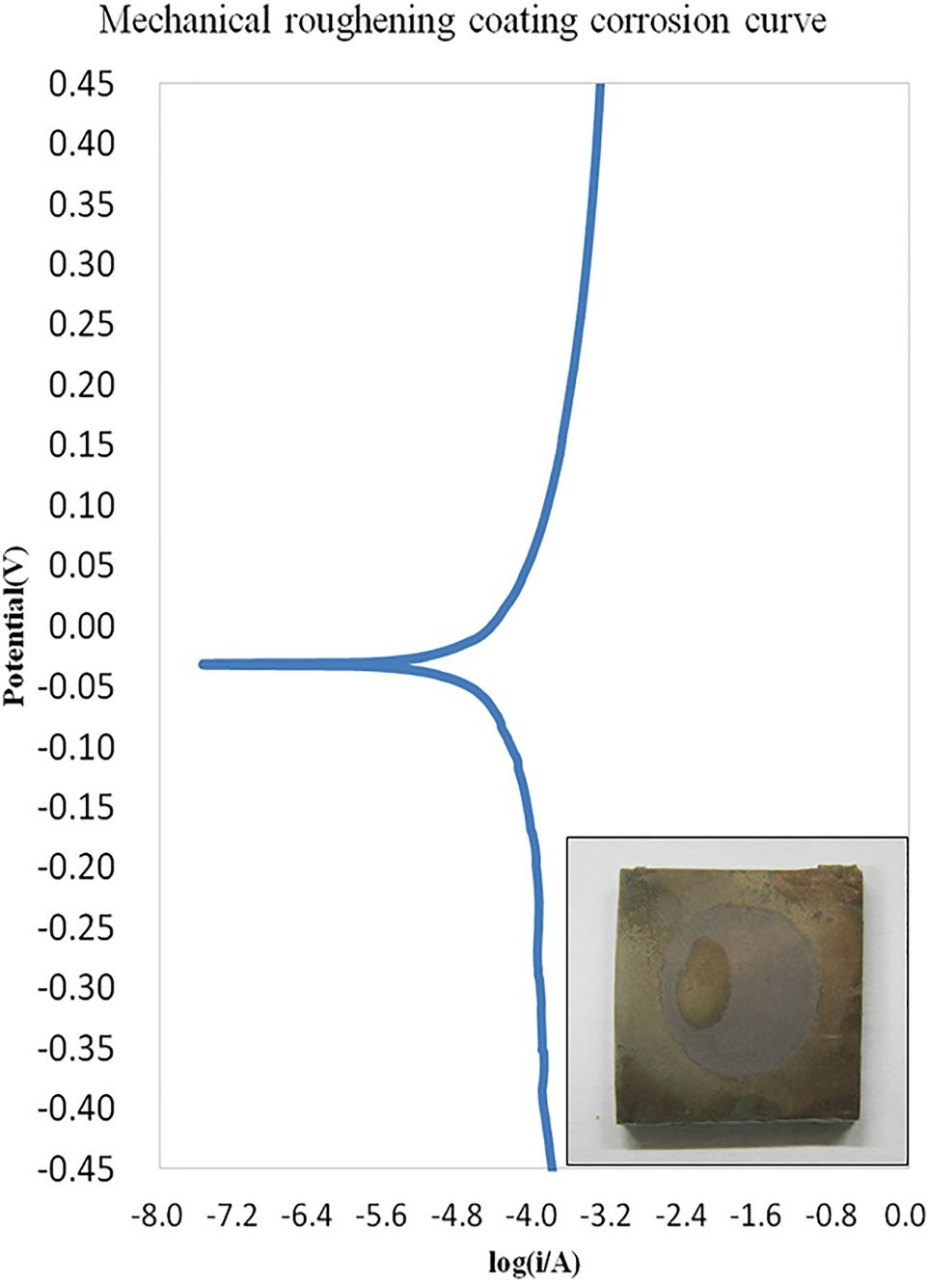
Electrochemical corrosion of Gold-coated on PMMA bipolar plate.

Bipolar plate is the key component of PEMFC which biggest problem of bipolar plate in PEMFC environment is corrosion, which may cause many adverse effects on bipolar plate and battery. Therefore, this study carried out electrochemical corrosion analysis with high accuracy and sensitivity of metal-based PMMA bipolar plate. The corrosion potential (Ecorr) and corrosion current density (jcorr) were extracted form Tafel curves (Fig. 12) to assess anti-corrosion performance of metal-based PMMA bipolar plate. This study investigated the effect of surface modification on the coating PMMA sheet and the effects of corrosion and the corrosion mechanism using an electrochemical gold plating film under a simulated fuel cell environment corrosion rate. Figure 12 shows that the mechanical roughening gold plating film has a corrosion potential of − 0.031 V and a corrosion current of 2.533 × 10^−5^ A. However, uncoated PMMA bipolar plates has a corrosion potential of − 0.031 V and a corrosion current of 2.533 × 10^−5^ A. Higher corrosion potential means less corrosion, while lower corrosion current means slower corrosion rate. The results show that PMMA bipolar plate with metal coating process has good corrosion resistance. This is due to the low activity of Au, not easy to oxidize and good corrosion resistance. Therefore, a 1 µm gold coating of metal-based PMMA bipolar plate was used as the anti-corrosion condition in this study.

### Electrical analysis of PMMA bipolar plate

**Figure 13:**
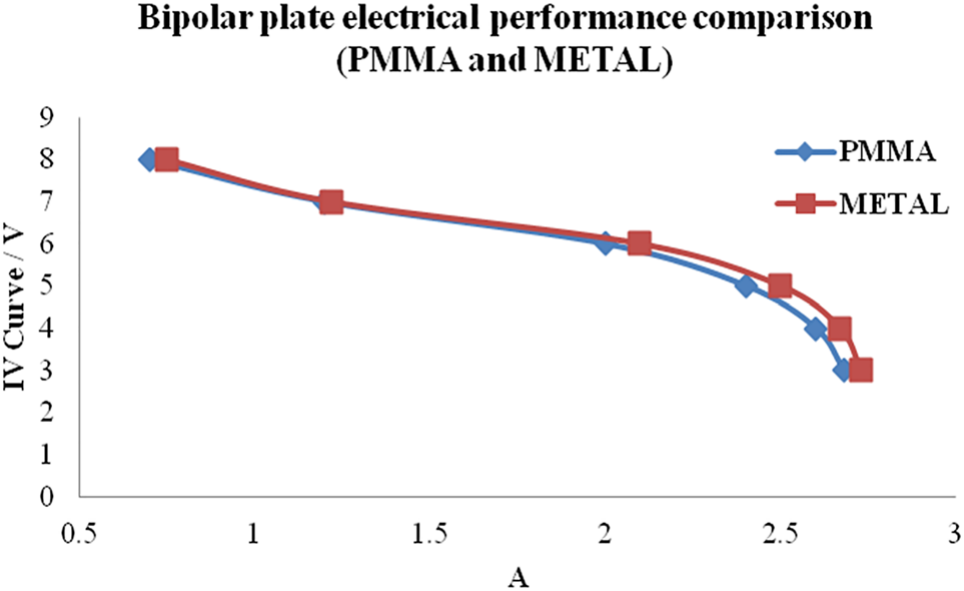
Comparison of electrical properties between PMMA bipolar plate and metal-coated PMMA bipolar plate.

Metal-based PMMA bipolar plate made from batteries were used to compare the difference in the electrical resistance of the modified PMMA composite bipolar plate metal film and the general conventional metallic bipolar plate. The fuel used was hydrogen at 99.999% purity, which is used for air-breathing. To assess the effect of the metal-based PMMA bipolar plate coating on the PEMFC performance, the current density on the output cell voltage and the power density for single PEMFC cell using uncoated or coated PMMA bipolar plates were explored. The PMMA composite bipolar plate and the metal bipolar plate battery power were compared as shown in Fig. 13. With metal‐coated PMMA bipolar plates on both anode and cathode side, the current density of a single cell at 0.6 V was found to be 2.1 A/cm^2^, which is better than uncoated PMMA bipolar plates (1.98 A/cm^2^).Generally, the out voltage cell decreased with current density increasing due to IR drop. This confirms that the using of metal-based PMMA bipolar plate leads to increase the performance of PEMFC cell. The above results reveal the strong effect of metal-based PMMA bipolar plate on the anti-corrosion effect of metal coating for PMMA bipolar plates, leading to an increased PEMFC performance.

**Figure 14:**
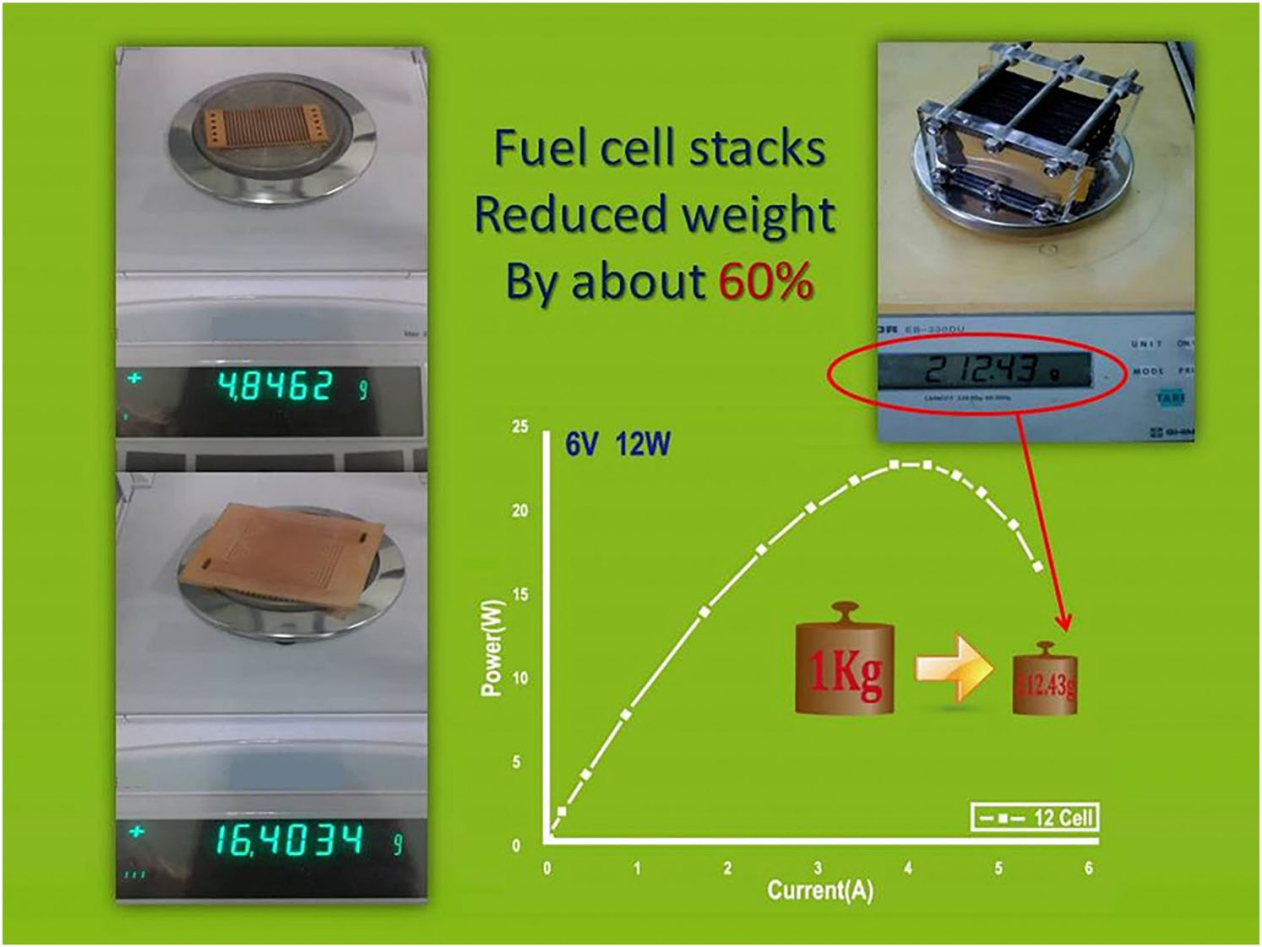
Weight of metal-coated PMMA battery system.

The battery system weight from the conventional metal bipolar plates was set to about 1 kg to 212 g, as shown in Fig. 14. In this study, a PEMFC with light weight, good corrosion resistance and good electrical performance was successfully developed.

## Conclusions

This research focuses on reducing the overall weight and volume of bipolar plate and increasing the adhesion of bimetallic materials by using metal coating. Metal-based PMMA bipolar plate was successfully applied to the development of PEMFC. The results of this research and future development plans are as follows:

- CAE simulation helps to design the plastic injection molding of bipolar plates, thereby improving mold development and reducing the cost of repairing molds. In this study, the optimal inlet position and the stomata position was the side entrance.
- Surface metallization adhesion is greater than 50 N when the temperature used is above 80 °C. Therefore, a temperature above 80 °C is a suitable working temperature for the PEMFC system.
- In the future, different plastic material injection molding approaches and cladding layers will be tested. Curved bipolar plates and leak-proof methods will also be developed to extend the bipolar plate bonding technology.
- The problem of conventional weight is solved via a weight reduction of 70%. 6 V–12 W battery systems, which are only 212 g, were used.
- Breakthroughs in the combination techniques for polymer and surface conductive layers were achieved. The conductive surfaces and lines and the cell stack were based on different voltage and current designs.
- The system can be mass produced, which substantially reduces overall costs. The production cost of the bipolar plates will decrease from $42 to $15, a 70% savings (USD).
- In the next stage, the innovative structure of the fuel cell bipolar plate in this research will be integrated with a flight system. The fuel cell will be used as a generator to provide the main power, supply the battery for energy storage, and drive the motor.
